# FAT10 Combined with Miltefosine Inhibits Mitochondrial Apoptosis and Energy Metabolism in Hypoxia-Induced H9C2 Cells by Regulating the PI3K/AKT Signaling Pathway

**DOI:** 10.1155/2022/4388919

**Published:** 2022-08-18

**Authors:** Yi Yao, Weikun Jia, Xiaofei Zeng, Yali Wang, Qiuxia Hu, Shiran Yu, Dongsheng He, Ying Li

**Affiliations:** ^1^Department of Cardiothoracic Surgery, The First People's Hospital of Neijiang, Neijiang, China; ^2^Department of Cardiothoracic Surgery, The first Affiliated Hospital of Chengdu Medical College, Chengdu 610500, China; ^3^Department of Cardiovascular Surgery, Affiliated Hospital of North Sichuan Medical College, Nanchong 637000, China

## Abstract

Hypoxia-induced cardiomyocyte apoptosis is the main contributor to heart diseases. Human leukocyte antigen F-associated transcript 10 (FAT10), the small ubiquitin-like protein family subtype involved in apoptosis, is expressed in the heart and exhibits cardioprotective functions. This study explored the impact of FAT10 on hypoxia-induced cardiomyocyte apoptosis and the involved mechanisms. The cardiomyocyte cell line H9C2 was cultivated in hypoxia-inducing conditions (94% N_2_, 5% CO_2,_ and 1% O_2_) and the expression of FAT10 in hypoxia-stimulated H9C2 cells was identified. For this, FAT10 overexpression/interference vectors were exposed to transfection into H9C2 cells with/without the PI3K/AKT inhibitor, miltefosine. The results indicated that hypoxia exposure decreased the FAT10 expression, suppressed H9C2 cell growth, disrupted mitochondrial metabolism, and promoted H9C2 cell apoptosis and oxidative stress. However, these impacts were reversed by the FAT10 overexpression. In addition, the inhibition of PI3K/AKT in FAT10-overexpressing cells suppressed cell proliferation, impaired mitochondrial metabolism, and promoted apoptosis and oxidative stress response. The findings demonstrated that FAT10 inhibited mitochondrial apoptosis and energy metabolism in hypoxia-stimulated H9C2 cells through the PI3K/AKT pathway. This finding can be utilized for developing therapeutic targets for treating heart disorders associated with hypoxia-induced apoptosis.

## 1. Introduction

Hypoxia is a prevalent pathophysiological event in cardiovascular diseases. Inadequate oxygen supply hinders energy metabolism in cardiomyocytes, leading to cell injury and reduced cardiac functional ability [1]. Mitochondrial damage occurring in cardiomyocytes upon hypoxic exposure induces apoptosis together with oxidative stress (OS), References [2, 3]. Hypoxia-induced cardiomyocyte apoptosis is the major contributor to heart disorders, including myocardial infarction [4, 5] and congenital heart disease, Reference [6]. Because cardiomyocytes are terminally differentiated, preventing their loss is the major challenge in treating heart diseases [7]. Another challenge is the complicated mechanism of hypoxia-induced cardiomyocyte apoptosis references [2]. Consequently, investigating molecular mechanisms underlying hypoxia-induced cardiomyocyte apoptosis can contribute to developing potential therapeutic strategies.

Human leukocyte antigen F-associated transcript 10 (FAT10), the small ubiquitin-like protein family subtype, is upregulated in several tumor types, including hepatocellular carcinoma (HCC), gastric carcinoma, and gynecological malignancies [8]. Additionally, FAT10 is expressed in the heart and exerts a cardioprotective effect. Reference [9] reported an enhancement in FAT10 levels in cultivated neonatal rat cardiomyocytes that experienced a hypoxia or reoxygenation condition. The elevated FAT10 level caused an enhancement in the level of B cell leukemia or lymphoma 2, also known as apoptosis regulator Bcl-2 (Bcl-2), and a decrease in the level of BCL2 associated X protein (Bax), thereby inhibiting hypoxia- or reoxygenation-induced cardiomyocyte apoptosis [10]. Furthermore, previous research studies have suggested that FAT10 regulates several cellular processes through protein kinase B (AKT) and AKT-related signaling pathways. Zou demonstrated that FAT10 contributed to bladder cancer development by promoting EGFR/AKT-mediated activation of HK2 [11], which suggested that FAT10 induced epithelial-mesenchymal transition and accelerated HCC cell metastasis by modulating the AKT/GSK3*β* pathway, thereby affecting the HCC prognosis [12]. Moreover, AKT refers to a downstream effector of phosphoinositide 3-kinase (PI3K); therefore, PI3K/AKT represents a critical pathway of cellular processes that are responsible for cell survival [13, 14]. A study indicated that the activation of PI3K/AKT signaling inhibited hypoxia-induced apoptosis in vascular endothelial cells [15]. Apart from that, the PI3K/AKT pathway was related to the apoptosis of cardiomyocytes subjected to ischemic hypoxia [4]. Nevertheless, the impact of FAT10 on hypoxia-induced cardiomyocyte apoptosis and whether its potential action mechanism is related to PI3K/AKT signaling remain unexplored.

In this study, the cardiomyocyte cell line H9C2 was cultured in hypoxic conditions to evaluate the expression of FAT10. The FAT10 overexpression or interference vectors were co-transfected into H9C2 cells with or without the PI3K/AKT inhibitor, miltefosine. Cells were exposed to treatment with 1 *μ*M miltefosine for 48 h [16], followed by culturing in hypoxic conditions to study the impact of FAT10 on hypoxia-induced cardiomyocyte apoptosis.

## 2. Materials and Methods

### 2.1. Cell Culture Procedures

The cardiomyocyte H9C2 cells (Shanghai Institutes for Biological Sciences, Chinese Academy of Science) were cultivated in Dulbecco's Modified Eagle Medium (DMEM)-high glucose medium (^#^SH30022.01, Hyclone, USA) containing 10% fetal bovine serum (FBS, ^#^10270–106, Gibco, USA) with 5% CO_2_ as well as 95% O_2_ at 37°C. In terms of hypoxia induction, H9C2 cells at the logarithmic growth phase were cultured at 37°C with 5% CO_2,_ 1% O_2_, and 94% N_2_ for 6, 12, and 24 h. Cell viability was detected, and the FAT10 expression was determined with the application of the Cell Counting kit-8 (CCK-8) assay using quantitative RT-PCR (qRT-PCR).

The H9C2 cell suspension was seeded into a 12-well plate at 1 × 10^5^ cells per well, and cells were transfected once the cell density reached 70%. Subsequently, 1.6 *μ*g of the FAT10 overexpression or interference plasmids were diluted with 100 *μ*L opti-MEM (^#^31985070, Gibco, USA). Besides, the mixture was incubated for 5 min and mixed with the transfection reagent containing 2 *μ*L lipofectamine 2000 reagent (^#^11668027, Invitrogen, USA) and 100 *μ*L opti-MEM; the mixture was incubated for 20 min. Next, 800 *μ*L of the basic culture medium and 200 *μ*L plasmid mixtures were supplemented to each well of the 12-well plate and incubated at 37°C with 5% CO_2_ for 48 h.

To evaluate cell apoptosis, oxidative stress, and mitochondrial metabolism, cells were cultured for 24 h based on hypoxic conditions (5% CO_2,_ 1% O_2_, and 94% N_2_) with the presence or absence of 1 *μ*M miltefosine (^#^S3056, Selleck, USA), an inhibitor of PI3K/AKT.

### 2.2. Cell Activity

The CCK-8 assay was carried out with the purpose of examining H9C2 cell viability. For this, H9C2 cells (3 × 10^3^/well) were seeded in a 96-well plate, followed by overnight incubation with 5% CO_2_ and 95% O_2_ at 37°C. Cells were exposed to transfection with the FAT10 overexpression or interference plasmids and with or without 1 *μ*M miltefosine, followed by culturing at hypoxic conditions for 24 h (5% CO_2,_ 1% O_2_, and 94% N_2_). Thereafter, CCK-8 (10 *μ*L, ^#^CA1210, Solarbio, China) was supplemented to each well and further cultured for 4 h. Besides, the plate was placed on a microplate reader (Allsheng, China), and the optical density was read at 450 nm.

### 2.3. mRNA Expression

The levels of FAT10 mRNA in hypoxia-stimulated and transfected H9C2 cells were measured using qRT-PCR. For this, the extraction of H9C2 RNA was performed by adopting Trizol Reagent (^#^15596–018, Invitrogen, USA), and DNA was eliminated using DNase I (^#^EN0521, Fermentas, USA). RNA was converted into cDNA with the application of a reverse transcription kit (^#^639505, TAKARA, Japan) according to the manufacturer's instructions. PCR amplification with a SYBR Green PCR kit (^#^KM4101, KAPA Biosystems, USA) was conducted based on the manufacturer's instructions. The primers are FAT10, forward (F), 5′-CCTCAAGCCCCATAGA-3′, reverse (R), 5′-GGCACAGCAGTCACATT-3'; GAPDH (housekeeping control), F, 5′-CAAGTTCAACGGCACAG-3′, R, 5′-CCAGTAGACTCCACGACAT-3'. The 2^–ΔΔCT^ formula was utilized for data analysis.

### 2.4. Cell Apoptosis

H9C2 cell apoptosis was identified with the use of the annexin V-fluorescein isothiocyanate (FITC) or propidium iodide (PI) apoptosis detection kit (^#^556547, BD Biosciences, USA). Subsequently, 1 × 10^6^ H9C2 cells were resuspended in DMEM, followed by 5 min centrifugation at 4°C and 400 × *g*. PBS (1 ml) was supplemented to the resuspended cells, followed by 5 min centrifugation at 4°C and 400 × *g*. Cells were resuspended in PBS (200 *μ*L), followed by 30 min staining with annexin V-FITC (10 *μ*L) as well as PI (10 *μ*L) in the dark at 4°C. After the addition of PBS (100 *μ*L), cells were detected using flow cytometry (ACEA Biosciences, USA).

### 2.5. OS-Related Factor Levels and Mitochondrial Metabolism

The levels of malondialdehyde (MDA), superoxide dismutase (SOD), lactic dehydrogenase (LDH), glutathione (GSH), and glutathione peroxidase (GSH-PX) were detected using commercially available kits (^#^A020–1–2, ^#^A003–1–1, ^#^A005–1–2, ^#^A006–1–1; Nanjing Jiancheng Bioengineering Institute, China) to investigate OS reaction in treated H9C2 cells. Similarly, the levels of glucose (^#^F006–1–1), lactic acid (^#^A019–2–1), and adenosine triphosphate (ATP, ^#^A095–1–1; Nanjing Jiancheng Bioengineering Institute, China) were also detected by evaluating mitochondrial metabolism in treated H9C2 cells. The instructions of protocols were followed.

### 2.6. Reactive Oxygen Species (ROS) Production

Cells expressing increased levels of ROS were detected based on the ROS Assay Kit (^#^S0033S, Beyotime, China). For this, 1 × 10^6^ H9C2 cells were resuspended in 1 mL of diluted 10 *μ*mol/L 2′-7′dichlorofluorescin diacetate (DCFH-DA), followed by 20 min incubation with 5% CO_2_ and 95% O_2_ at 37°C. In addition, cells were resuspended in PBS (500 *μ*L), followed by detection with flow cytometry (FCM, ACEA Biosciences, USA).

### 2.7. Mitochondrial Membrane Potential (MMP)

Cells with a reduced MMP were measured using the JC-1 detection kit (^#^C2006; Beyotime, China). For this, 1 × 10^6^ H9C2 cells were resuspended in DMEM (500 *μ*L), followed by 30 min staining by using JC-1 (500 *μ*L) and 3 min centrifugation at 400 × *g* at 4°C. Cells were resuspended in JC-1 (1 ml), followed by 3 min centrifugation at 400 × *g* and 4°C; cells were resuspended again in JC-1 (400 *μ*L). Then, cells were determined using flow cytometry (ACEA Biosciences, USA).

### 2.8. Observation of the Mitochondrial Ultrastructure

The mitochondrial ultrastructure of H9C2 cells was observed with the transmission electron microscope. For this, 1 × 10^7^ H9C2 cells were prefixed for 20 min with 2 mL of 2.5% glutaraldehyde, followed by 1 h post-fixation by adding 1% osmic acid. Through dehydration, permeation, and embedding, the samples were sliced into 60 nm-thick samples, followed by 20 min staining with uranyl acetate and another 15 min staining with lead citrate in the dark. The mitochondrial ultrastructure was observed with the use of a transmission electron microscope (Hitachi).

### 2.9. Protein Expression

By employing Western blotting, the expression of apoptotic proteins and PI3K/AKT pathway activation was evaluated. The extraction of whole proteins was performed from H9C2 cells by using the RIPA lysis buffer (^#^R0010, Solarbio, China); proteins were quantified by using a BCA assay kit (^#^PC0020, Solarbio, China). Subsequently, 20 *μ*g of proteins from each group were separated using SDS-PAGE, followed by their transfer onto polyvinylidene difluoride (PVDF) membranes. Apart from that, membranes were blocked using 5% defatted milk, followed by 1 h incubation with anti-mitofusin 2 (MFN2), anti-dynamin-related protein 1 (DRP1), anti-mitochondrial fission process 1 (MTFP1), anti-phosphorylation (p)-DRP1(S637), anti-p-DRP1 (S616), anti-Bcl-2, anti-Bax, anti-cytochrome C (Cyt-c), anti-FAT10, anti-PI3K, anti-p-PI3K, anti-AKT, and anti-p-AKT, together with anti-*β*-actin (housekeeping control) primary antibodies. Next, membranes were further incubated with HRP-conjugated goat anti-rabbit IgG for an hour. All antibodies were obtained from Bioswamp. The primary antibodies were used at a dilution of 1 : 1000, whereas the dilution of the secondary antibodies was 1 : 20,000. Signals were identified with the automatic imaging system (Tanon-5200, Tanon, China).

### 2.10. Statistical Analysis

Data are denoted to be mean ± SD. One-way analysis of variance (ANOVA) in combination with the Tukey test was employed with the aim of analyzing differences across diverse groups; *p* < 0.05 was regarded to show statistical significance. GraphPad Prism 8 software was employed for graph construction.

## 3. Results

### 3.1. Hypoxia Downregulates the FAT10 Expression in H9C2 Cells

**Figure 1:**
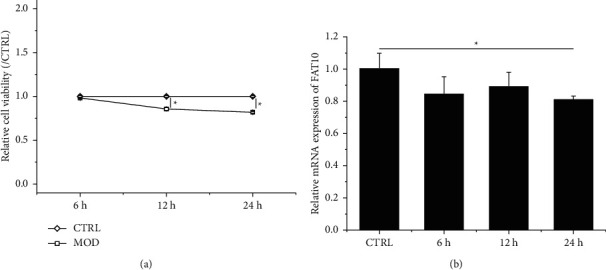
Hypoxia inhibits H9C2 cell proliferation and downregulates FAT10 expression. (a) Viability assessment of hypoxia-stimulated H9C2 cells with CCK-8. (b) Evaluation of FAT10 mRNA levels in hypoxia-stimulated H9C2 cells with qRT-PCR. Data are denoted to be mean ± SD, *n* = 3, ^*∗*^*p* < 0.05.

According to Figures 1(a) and 1(b), hypoxia suppressed H9C2 cell viability and downregulated FAT10 mRNA levels through the time-dependent manner. In comparison with control cells, a statistical difference in FAT10 mRNA expression was observed after 24 h of hypoxic culturing; therefore, we considered 24 h as the culture time for further experiments.

### 3.2. Effect of the FAT10 Overexpression on Proliferation of Hypoxia-Stimulated H9C2 Cells and Apoptosis Reversion through PI3K/AKT Inhibition

**Figure 2:**
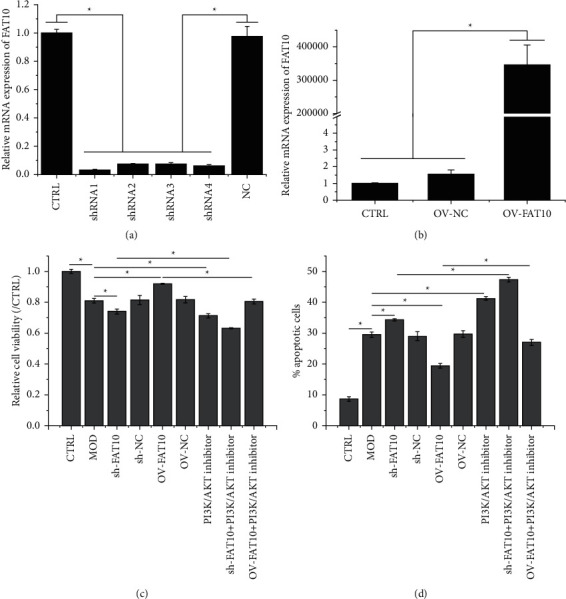
Effects of the FAT10 overexpression and PI3K/AKT inhibition on hypoxia-mediated H9C2 cell proliferation and apoptosis. Detection of FAT10 mRNA expression by using qRT-PCR in H9C2 cells transfected with (a) interference or (b) overexpression vectors. (c) Measurement of H9C2 cell viability by using CCK-8. (d) Determination of apoptotic H9C2 cells with the application of flow cytometry. Data are denoted to be mean ± SD, *n* = 3, ^*∗*^*p* < 0.05.

**Figure 3:**
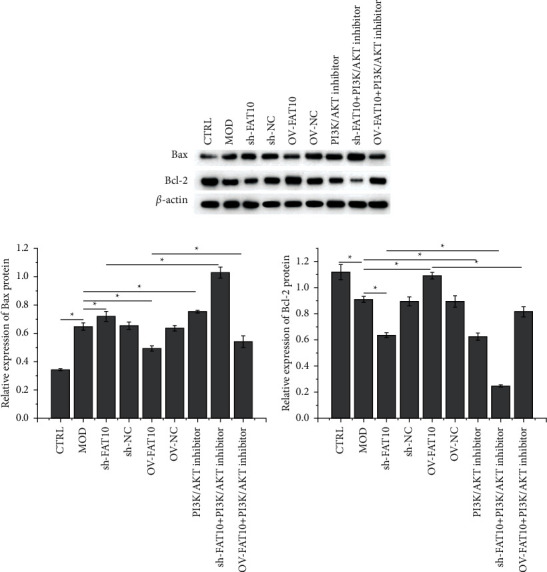
Effects of the FAT10 overexpression and PI3K/AKT inhibition on hypoxia-mediated H9C2 cell apoptosis. Evaluation of apoptotic protein expression in H9C2 cells by adopting Western blot. Besides, data are denoted to be mean ± SD, *n* = 3, ^*∗*^*p* < 0.05.

As depicted in Figures 2(a) and 2(b), the FAT10 expression in H9C2 cells was silenced or overexpressed by interference and overexpression vectors, respectively, and shRNA1 was randomly chosen for the subsequent experiments. According to the CCK-8 assay, the FAT10 overexpression weakened the inhibitory effect of hypoxia in H9C2 cells, whereas this effect was strengthened by FAT10 interference or PI3K/AKT inhibition prior to the hypoxia treatment (Figure 2(c)). In addition, hypoxia-induced H9C2 cell apoptosis was accentuated by FAT10 interference or PI3K/AKT inhibition but weakened by FAT10 overexpression prior to the hypoxia treatment. Apoptosis in FAT10-overexpressing hypoxia-stimulated cells was further promoted by miltefosine treatment (Figure 2(d)). The apoptotic protein levels, analyzed through Western blot, revealed that hypoxia treatment upregulated Bax, and this effect was accentuated by FAT10 interference or PI3K/AKT inhibition but weakened by FAT10 overexpression prior to the hypoxia treatment. Bax protein levels in FAT10-overexpressing hypoxia-stimulated cells further increased after miltefosine exposure. The Bcl-2 expression exhibited the opposite trend as that of Bax (Figure 3).

### 3.3. Inhibitory Effect of the FAT10 Overexpression on Oxidative Stress of Hypoxia-Stimulated H9C2 Cells and Reversion by PI3K/AKT Inhibition

**Figure 4:**
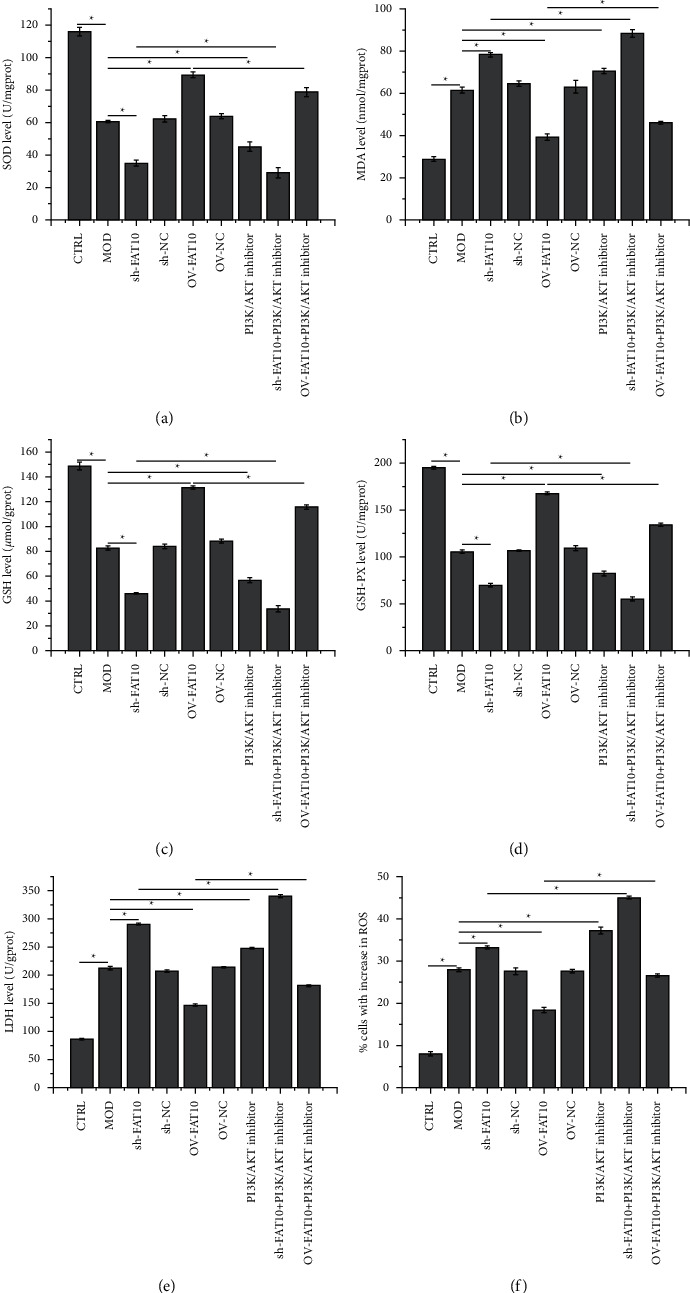
Effects of the FAT10 overexpression and PI3K/AKT inhibition on oxidative stress in hypoxia-stimulated H9C2 cells. Levels of (a) SOD, (b) MDA, (c) GSH, (d) GSH-PX, and (e) LDH in H9C2 cells. (f) Detection of H9C2 cells with enhanced ROS production by employing flow cytometry. Data are denoted to be mean ± SD, *n* = 3, ^*∗*^*p* < 0.05.

As depicted in Figure 4, hypoxia decreased SOD, GSH, and GSH-PX levels, and this effect was strengthened by FAT10 interference or PI3K/AKT inhibition but weakened by the FAT10 overexpression prior to the hypoxia treatment. The levels of SOD, GSH, and GSH-PX in FAT10-overexpressing hypoxia-stimulated cells decreased after miltefosine treatment. The levels of MDA and LDH and cell proportion with increased ROS production demonstrated an opposite trend as that of SOD, GSH, and GSH-PX levels.

### 3.4. Impact of the FAT10 Overexpression on Mitochondria-Mediated Energy Metabolism in Hypoxia-Stimulated H9C2 Cells and Reversion by PI3K/AKT Inhibition

**Figure 5:**
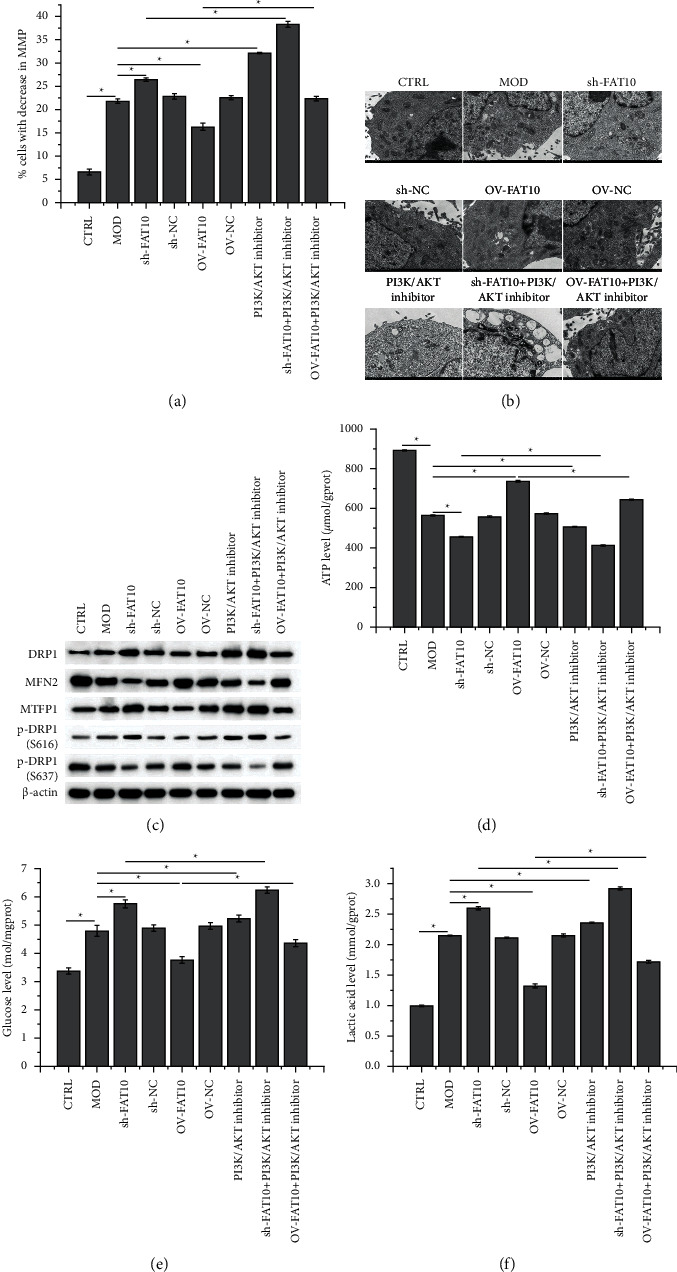
Effects of the FAT10 overexpression and PI3K/AKT inhibition on mitochondria-mediated energy metabolism in hypoxia-stimulated H9C2 cells. (a) Detection of H9C2 cells by using flow cytometry revealed decreased MMP levels. (b) Transmission electron microscopy images of mitochondrial ultrastructure in H9C2 cells. (c) Expression of proteins associated with mitochondrial fission and fusion in H9C2 cells by using Western blot. Levels of (d) ATP, (e) glucose, (f), and lactic acid in H9C2 cells. Data are represented to be mean ± SD, *n* = 3, ^*∗*^*p* < 0.05.

Hypoxia enhanced the number of cells with lowered MMP (Figure 5(a)) and the expressions of DRP1, MTFP1, p-DRP1 (S616), glucose (Figure 5(e)), and lactic acid (Figure 5(f)); decreased MFN2, p-DRP1 (S637) (Figure 5(c)), and ATP (Figure 5(d)) levels and disrupted the mitochondrial ultrastructure (Figure 5(b)). The effects of hypoxia were aggravated by FAT10 interference or PI3K/AKT inhibition but mitigated by FAT10 overexpression prior to the hypoxia treatment. In addition, these effects in FAT10-overexpressing hypoxia-stimulated cells were reversed by the addition of miltefosine.

### 3.5. FAT10 Overexpression Enhances PI3K/AKT Signal Activation

**Figure 6:**
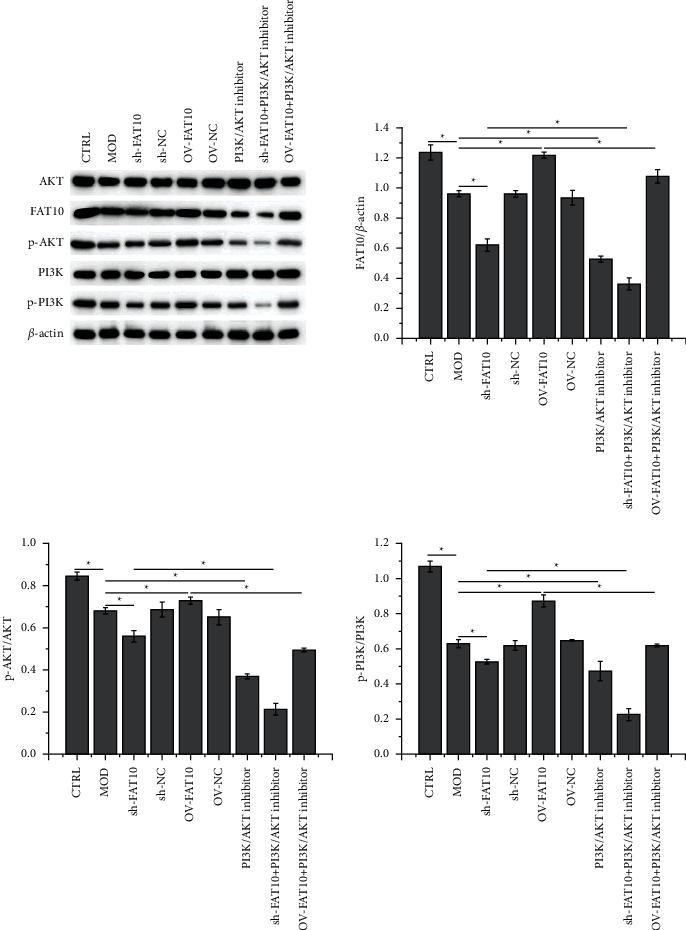
FAT10 overexpression promotes PI3K/AKT pathway activation. Evaluation of FAT10, AKT, p-AKT, PI3K, and p-PI3K protein levels in H9C2 cells by adopting Western blot. In addition, data are denoted to be mean ± SD, *n* = 3, ^*∗*^*p* < 0.05.

According to the results of Western blot, hypoxia treatment reduced FAT10, p-AKT, and p-PI3K activities, and this effect was strengthened by FAT10 interference or PI3K/AKT inhibition but weakened by FAT10 overexpression prior to the hypoxia treatment. The activities of FAT10, p-AKT, and p-PI3K in FAT10-overexpressing hypoxia-stimulated cells were suppressed by the addition of miltefosine (Figure 6).

## 4. Discussion

Apoptosis involves highly complicated mechanisms and a series of energy-dependent molecular events [17]. In general, the mechanism of apoptosis is classified into two primary pathways: the intrinsic pathway and the extrinsic pathway. The intrinsic pathway or mitochondrial apoptosis is a mitochondria-dependent process that induces apoptosis through intracellular signals that directly target cells; these signals are produced by various nonreceptor-mediated stimuli [17]. Mitochondria are an energy-producing organelle and a dominating ROS cell source. Mitochondria manage cell survival and apoptosis principally through fission and fusion, processes involved in homeostatic balance maintenance [18–20]. Several proteins that control mitochondrial fusion and fission, including optic atrophy 1, DRP1, MTFP1, and MFN1/2, are also implicated in apoptosis and are indispensable for maintaining mitochondrial morphology [21–24]. Mitochondrial ultrastructure and homeostasis are intimately associated with the crosslink between cell survival/death and bioenergetic processes [25]. Moreover, the mitochondrial ultrastructure directly influences the bioenergetic function of mitochondria [26]. Mitochondrial fragmentation is often associated with mitochondrial dysfunction and ATP depletion [26, 27]. Accumulating evidence indicates that inhibiting mitochondrial fusion enhances apoptosis. Proteins associated with mitochondrial fusion, namely, MFN1 and MFN2, not only increase mitochondrial connectivity but also leads to Bax activation and Cyt-c release delay, thereby inhibiting mitochondrial apoptosis [28]. A study reported that the inhibition of MFN1 or MFN2 increased the sensitivity of apoptotic stimuli, thereby leading to mitochondrial fragmentation [28]. Mitochondria is a primary source of ROS, whose accumulation contributes to OS [29, 30]. Under OS conditions, MFN2 expression declines, whereas the protein related to mitochondrial fission, DRP1, elevates, leading to mitochondrial fragmentation, decreased energy metabolism, and ultimately mitochondrial apoptosis [31–33]. Our results indicated that hypoxia enhanced OS response, promoted mitochondrial apoptosis, and inhibited the metabolism of mitochondrial energy in H9C2 cells; these findings conform to those of previous studies [34–37]. However, the effects of hypoxia were weakened by the FAT10 overexpression.

Pretreatment with miltefosine to inhibit PI3K/AKT aggravated hypoxia-induced damage in H9C2 cells and negated the attenuating impact of the FAT10 overexpression on hypoxia-induced H9C2 cell damage. Several studies have suggested that FAT10 participates in several cellular events through the regulation of AKT-related signaling pathways [11, 12]. AKT refers to a downstream effector for PI3K, and the PI3K/AKT signaling pathway modulates cellular events, including cell growth, metabolism, and apoptosis [38–40] demonstrated that PI3K/AKT activation attenuated OS-induced mitochondria-dependent apoptosis by upregulating Bcl-2 and suppressing Bax, Cyt-c, and cleaved caspase 9/3 expression [41]. PI3K/AKT pathway activation also inhibited OS and apoptosis in streptozotocin-mediated diabetic cardiomyopathy [42]. Apart from that, PI3K/AKT activation suppressed doxorubicin-induced mitochondrial OS by reducing ROS production in H9C2 cells and prevented doxorubicin-induced insufficient mitochondrial fusion and excessive mitochondrial fission by promoting DRP1 phosphorylation (S637) [43]. Miltefosine is the most commonly used PI3K/AKT pathway inhibitor [44].

## 5. Conclusion

In conclusion, our work revealed that FAT10 inhibited intrinsic apoptosis induced by OS and mitochondrial dysfunction in hypoxia-stimulated H9C2 cells. We suggest that the underlying mechanism is mediated by PI3K/AKT signaling activation, which was inhibited by miltefosine. These findings define FAT10 as the therapeutic candidate to treat heart disorders, including cyanotic congenital heart disease, pulmonary hypertension, cardiac hypertrophy, heart failure, and atherosclerosis, that are associated with hypoxia-induced apoptosis.

## Data Availability

All the data are included in the manuscript.

## References

[1] Yu H., Chen B., Ren Q. (2019). Baicalin relieves hypoxia-aroused H9c2 cell apoptosis by activating Nrf2/HO-1-mediated HIF1*α*/BNIP3 pathway. *Artificial Cells, Nanomedicine, and Biotechnology*.

[2] Chen Z., Zhang S., Guo C., Li J., Sang W. (2017). Downregulation of miR-200c protects cardiomyocytes from hypoxia-induced apoptosis by targeting GATA-4. *International Journal of Molecular Medicine*.

[3] Glinka Y. Y., Youdim M. B. (1995). Inhibition of mitochondrial complexes I and IV by 6-hydroxydopamine. *European Journal of Pharmacology: Environmental Toxicology and Pharmacology*.

[4] Shi K., Sun H., Zhang H., Xie D., Yu B. (2019). **miR-34a-5p** aggravates hypoxia-induced apoptosis by targeting ZEB1 in cardiomyocytes. *Biological Chemistry*.

[5] Xin T., Lu C. (2020). Irisin activates Opa1-induced mitophagy to protect cardiomyocytes against apoptosis following myocardial infarction. *Aging (Albany NY)*.

[6] Yang H., He X., Wang C., Zhang L., Yu J., Wang K. (2020). Knockdown of TUG 1 suppresses hypoxia-induced apoptosis of cardiomyocytes by upregulating miR-133a. *Archives of Biochemistry and Biophysics*.

[7] Meng X., Ji Y., Wan Z. (2017). Inhibition of miR-363 protects cardiomyocytes against hypoxia-induced apoptosis through regulation of notch signaling. *Biomedicine and Pharmacotherapy*.

[8] Lee C. G., Ren J., Cheong I. S. (2003). Expression of the FAT10 gene is highly upregulated in hepatocellular carcinoma and other gastrointestinal and gynecological cancers. *Oncogene*.

[9] Liu X., Ge J., Chen C. (2021). FAT10 protects against ischemia-induced ventricular arrhythmia by decreasing Nedd4–2/Nav1.5 complex formation. *Cell Death and Disease*.

[10] Peng X., Shao J., Shen Y. (2013). FAT10 protects cardiac myocytes against apoptosis. *Journal of Molecular and Cellular Cardiology*.

[11] Zou Y., Du Y., Cheng C. (2021). FAT10 promotes the progression of bladder cancer by upregulating HK2 through the EGFR/AKT pathway. *Experimental Cell Research*.

[12] Liu L., Dong Z., Liang J. (2014). As an independent prognostic factor, FAT10 promotes hepatitis B virus-related hepatocellular carcinoma progression via Akt/GSK3*β* pathway. *Oncogene*.

[13] Chen L., Monti S., Juszczynski P. (2013). SYK inhibition modulates distinct PI3K/AKT-dependent survival pathways and cholesterol biosynthesis in diffuse large B cell lymphomas. *Cancer Cell*.

[14] Yang J., Pi C., Wang G. (2018). Inhibition of PI3K/Akt/mTOR pathway by apigenin induces apoptosis and autophagy in hepatocellular carcinoma cells. *Biomedicine and Pharmacotherapy*.

[15] Shi C., Zhan L., Wu Y. (2020). Kaji-ichigoside F1 and rosamultin protect vascular endothelial cells against hypoxia-induced apoptosis via the PI3K/AKT or ERK1/2 signaling pathway. *Oxidative Medicine and Cellular Longevity*.

[16] Kindrachuk J., Ork B., Hart B. J. (2015). Antiviral potential of ERK/MAPK and PI3K/AKT/mTOR signaling modulation for Middle East respiratory syndrome coronavirus infection as identified by temporal kinome analysis. *Antimicrobial Agents and Chemotherapy*.

[17] Elmore S. (2007). Apoptosis: a review of programmed cell death. *Toxicologic Pathology*.

[18] Lane N., Martin W. (2010). The energetics of genome complexity. *Nature*.

[19] Westermann B. (2008). Molecular machinery of mitochondrial fusion and fission. *Journal of Biological Chemistry*.

[20] Polyakov V. Y., Soukhomlinova M. Y., Fais D. (2003). Fusion, fragmentation, and fission of mitochondria. *Biochemistry*.

[21] Otera H., Miyata N., Kuge O., Mihara K. (2016). Drp1-dependent mitochondrial fission via MiD49/51 is essential for apoptotic cristae remodeling. *Journal of Cell Biology*.

[22] Cipolat S., Rudka T., Hartmann D. (2006). Mitochondrial rhomboid PARL regulates cytochrome c release during apoptosis via OPA1-dependent cristae remodeling. *Cell*.

[23] Estaquier J., Vallette F., Vayssiere J. L., Mignotte B. (2012). The mitochondrial pathways of apoptosis. *Advances in Experimental Medicine and Biology*.

[24] Morita M., Prudent J., Basu K. (2017). mTOR controls mitochondrial dynamics and cell survival via MTFP1. *Molecular Cell*.

[25] Yamaguchi R., Perkins G. (2009). Dynamics of mitochondrial structure during apoptosis and the enigma of Opa1. *Biochimica et Biophysica Acta (BBA)—Bioenergetics*.

[26] Chan D. C. (2020). Mitochondrial dynamics and its involvement in disease. *Annual Review of Pathology: Mechanisms of Disease*.

[27] Brooks C., Wei Q., Cho S. G., Dong Z. (2009). Regulation of mitochondrial dynamics in acute kidney injury in cell culture and rodent models. *Journal of Clinical Investigation*.

[28] Suen D. F., Norris K. L., Youle R. J. (2008). Mitochondrial dynamics and apoptosis. *Genes and Development*.

[29] Poprac P., Jomova K., Simunkova M., Kollar V., Rhodes C. J., Valko M. (2017). Targeting free radicals in oxidative stress-related human diseases. *Trends in Pharmacological Sciences*.

[30] Jung H. S., Lee J. H., Kim K. (2017). A mitochondria-targeted cryptocyanine-based photothermogenic photosensitizer. *Journal of the American Chemical Society*.

[31] Wu N. N., Tian H., Chen P., Wang D., Ren J., Zhang Y. (2019). Physical exercise and selective autophagy: benefit and risk on cardiovascular health. *Cells*.

[32] Liang X., Wang S., Wang L., Ceylan A. F., Ren J., Zhang Y. (2020). Mitophagy inhibitor liensinine suppresses doxorubicin-induced cardiotoxicity through inhibition of Drp1-mediated maladaptive mitochondrial fission. *Pharmacological Research*.

[33] Wang Z., Sun R., Wang G. (2020). SIRT3-mediated deacetylation of PRDX3 alleviates mitochondrial oxidative damage and apoptosis induced by intestinal ischemia/reperfusion injury. *Redox Biology*.

[34] Tang Q., Li M. Y., Su Y. F. (2018). Absence of miR-223–3p ameliorates hypoxia-induced injury through repressing cardiomyocyte apoptosis and oxidative stress by targeting KLF15. *European Journal of Pharmacology*.

[35] Yan D., Tang B., Yan L. (2019). Sodium selenite improves the therapeutic effect of BMSCs via promoting the proliferation and differentiation, thereby promoting the hematopoietic factors. *OncoTargets and Therapy*.

[36] Fan J. L., Zhu T. T., Xue Z. Y. (2020). lncRNA-XIST protects the hypoxia-induced cardiomyocyte injury through regulating the miR-125b-hexokianse 2 axis. *In Vitro Cellular and Developmental Biology—Animal*.

[37] Xie H., Xu G., Gao Y., Yuan Z. (2020). hCINAP serves a critical role in hypoxiainduced cardiomyocyte apoptosis via modulating lactate production and mitochondrialmediated apoptosis signaling. *Molecular Medicine Reports*.

[38] Cheng J., Huang Y., Zhang X. (2020). TRIM21 and PHLDA3 negatively regulate the crosstalk between the PI3K/AKT pathway and PPP metabolism. *Nature Communications*.

[39] Liu L., Huang Y., Feng X., Chen J., Duan Y. (2019). Overexpressed Hsp70 alleviated formaldehyde-induced apoptosis partly via PI3K/Akt signaling pathway in human bronchial epithelial cells. *Environmental Toxicology*.

[40] Chai C., Song L. J., Han S. Y., Li X. Q., Li M. (2018). Retracted: micro RNA ‐21 promotes glioma cell proliferation and inhibits senescence and apoptosis by targeting SPRY 1 via the PTEN/PI 3K/AKT signaling pathway. *CNS Neuroscience and Therapeutics*.

[41] Zhao Q., Li H., Chang L. (2019). Qiliqiangxin attenuates oxidative stress-induced mitochondrion-dependent apoptosis in cardiomyocytes *via* PI3K/AKT/GSK3*β* signaling pathway. *Biological and Pharmaceutical Bulletin*.

[42] Ren B. C., Zhang Y. F., Liu S. S. (2020). Curcumin alleviates oxidative stress and inhibits apoptosis in diabetic cardiomyopathy via Sirt1-Foxo1 and PI3K-Akt signalling pathways. *Journal of Cellular and Molecular Medicine*.

[43] Li L., Li J., Wang Q. (2020). Shenmai injection protects against doxorubicin-induced cardiotoxicity via maintaining mitochondrial homeostasis. *Frontiers in Pharmacology*.

[44] Wang X. Y., Pan J. Y., Liu D. (2019). Nicorandil alleviates apoptosis in diabetic cardiomyopathy through PI3K/Akt pathway. *Journal of Cellular and Molecular Medicine*.

